# Partial anomalous pulmonary venous connection with accessory pulmonary veins

**DOI:** 10.5830/CVJA-2017-022

**Published:** 2018

**Authors:** Arulselvam Vimalarani, N Kalis Neale, R Al Amer Suad

**Affiliations:** Mohammed bin Khalifa bin Salman Al–Khalifa Cardiac Centre, Bahrain Defense Forces Hospital, Kingdom of Bahrain; Mohammed bin Khalifa bin Salman Al–Khalifa Cardiac Centre, Bahrain Defense Forces Hospital, Kingdom of Bahrain; Mohammed bin Khalifa bin Salman Al–Khalifa Cardiac Centre, Bahrain Defense Forces Hospital, Kingdom of Bahrain

**Keywords:** partial anomalous pulmonary venous connections, accessory pulmonary veins

## Abstract

We present a case of a six–year–old boy with complex partial anomalous pulmonary venous connections with accessory pulmonary veins, where multi–detector computed tomography proved crucial for accurate identification prior to planning for surgical correction.

Partial anomalous pulmonary venous connection is a rare congenital cardiovascular condition in which some but not all of the pulmonary veins drain into the systemic circulation rather than into the left atrium. Although the pulmonary venous anatomy can be evaluated by echocardiography and cardiac catheterisation, non–invasive modalities such as multi–detector computed tomography and magnetic resonance imaging now play a crucial role in characterisation of the pulmonary veins. We report on a case of partial anomalous pulmonary venous connection of the left superior pulmonary vein with bilateral accessory pulmonary veins.

## Case report

A six–year–old child who underwent aortic coarctation repair at two years of age was referred to us. He was asymptomatic and weighed 23 kg, with normal oxygen saturation in room air. There was no significant limb blood pressure gradient between the upper and lower limbs. His left radial and brachial pulses were absent. The cardiovascular examination revealed a grade 2/6 systolic murmur.

Chest X–ray showed mild cardiomegaly. Electrocardiography revealed right atrial and right ventricular enlargement. Echocardiography confirmed a dilated right atrium, ventricle and pulmonary arteries. The estimated right ventricular systolic pressure was 45 mmHg. There was a 20–mmHg gradient across the descending aorta. Evaluation of the pulmonary veins showed two right–sided veins draining normally into the left atrium and one left–sided pulmonary vein connecting to the vertical vein and draining into a dilated innominate vein and superior vena cava.

**Fig. 1.**
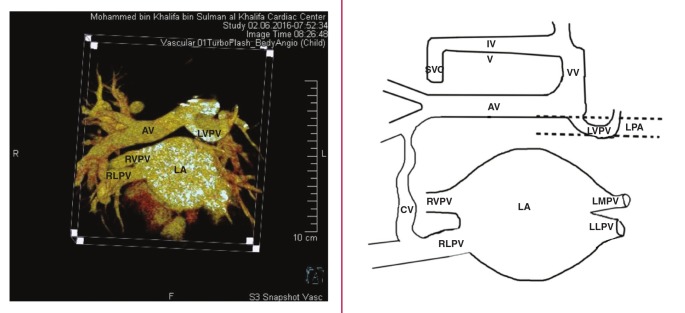
CT angiogram (anterior view). Normal drainage of the right upper (RUPV) and right lower pulmonary vein (RLPV) into the upper pole of the left atrium (LA), and a small left middle pulmonary vein (LMPV) and left lower pulmonary veins (LLPV) into the left atrium. Large right–sided accessory pulmonary vein (AV) drains into the right upper lobe of the lung. Left upper pulmonary vein (LUPV) makes a U–turn around the left pulmonary artery (LPA) and joins with the anomalous right accessory pulmonary vein draining into the vertical vein (VV). Right–sided anomalous accessory pulmonary vein also connects (CV) with the RLPV.

Multi–detector computed tomography (Fig. 1A, B) confirmed normal drainage of the right upper and lower pulmonary veins, a small left middle pulmonary vein, and left lower pulmonary veins draining into the upper poles of the left atrium.

**Fig. 2.**
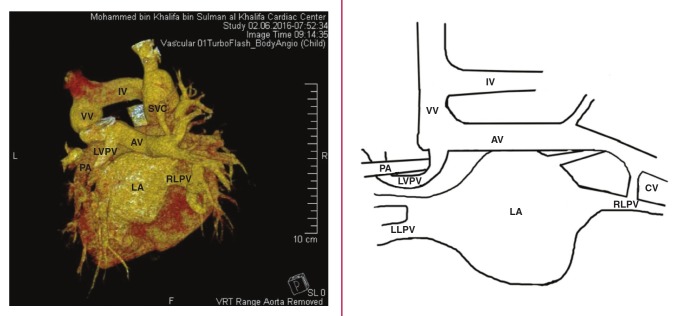
CT angiogram (posterior view). The left upper pulmonary vein (LUPV), making a U–turn around the left pulmonary artery (LPA), joins the accessory pulmonary vein (AV), which drains via a dilated vertical vein (VV) into the innominate vein (IV) and finally into the dilated right–sided superior vena cava (SVC). RLPV, right lower pulmonary vein; LA, left atrium; LLPV, left lower pulmonary vein; CV, connecting vein.

A large right–sided accessory pulmonary vein drained from the right upper lobe lung. This accessory pulmonary vein was dilated and had a long superior course to the left side of the heart before joining the left upper pulmonary vein, which made a U–turn around the left pulmonary artery. After joining, both drained superiorly into the innominate vein via a dilated vertical vein, which drained into the dilated right–sided superior vena cava (Fig. 2A, B). Furthermore, the lower branch of this rightsided anomalous accessory pulmonary vein was connected to the right lower pulmonary vein (Fig. 1A, B).

The patient was scheduled for surgical redirection of the anomalous pulmonary venous drainage to the left atrium.

## Discussion

The typical pattern of four pulmonary veins with welldifferentiated ostia is seen in 60 to 70% of the population.^1^ A typical anatomical patterns are found in approximately 38% of the population,^2^ hence it is important to be familiar with them.

The prevalence of partial anomalous pulmonary venous connections is 0.4 to 0.6%.^3^ Patients with partial anomalous pulmonary venous connections are often asymptomatic and are detected incidentally. If the anomaly compromises 50% or more of the pulmonary venous flow, it may become clinically significant.

Various normal patterns and variations have been described in studies of pulmonary vein anatomy.^1^,^2^ Anatomical variants on the left side are relatively simple, basically consisting of convergence of the left pulmonary veins into a common trunk that drains into the left atrium. Two subtypes of this variant occur: a short or a long left common trunk. The short left common trunk is the second most common normal anatomical pattern, occurring in 15% of the population.

Anatomical variants on the right side are less common and more complex, with one or more accessory veins that have their own connections to the left atrium independently of the superior and inferior pulmonary veins. These variants mainly include (1) one accessory right middle pulmonary vein, (2) two accessory right middle pulmonary veins, and (3) one accessory right middle pulmonary vein and one accessory right upper pulmonary vein.

Other infrequent variations are also seen: a superior segment right lower lobe vein, basilar segments of the right lower lobe, and a right upper pulmonary vein. A right upper pulmonary vein enters the left atrium at a point super–medial to the right superior pulmonary vein and drains into the superior right lower lobe segment, the posterior right upper lobe segment, or both segments.^1^,^4^

In our case, even though four pulmonary veins drained into the left atrium, one accessory pulmonary vein on the right side had dual drainage into the superior vena cava via the vertical vein and into the left atrium via a tortuous connection to the right inferior pulmonary vein. Also there was anomalous connection of the left superior pulmonary vein into the vertical vein.

Our case is unique where anomalous drainage from both upper lobe lungs contributed to approximately 66% of the pulmonary blood flow and needed to be corrected surgically. Multi–detector computed tomography proved crucial for accurate identification. To our knowledge there are no published reports with a similar condition.

## Conclusion

Echocardiography is the initial imaging technique of choice but it is sub–optimal in the complete evaluation of complex pulmonary venous anomalies. Multi–detector computed tomography provides very rapid, safe imaging that may obviate the need for sedation. Axial and three–dimensional reconstructed images accurately depict the anomalous pulmonary venous structures prior to further surgical management.

## References

[1] Lacomis JM, Goitein O, Deible C, Schwartzman D (2007). CT of the pulmonary veins. J Thorac Imaging.

[2] Kato R, Lickfett L, Meininger G (2003). Pulmonary vein anatomy in patients undergoing catheter ablation of atrial fibrillation: lessons learned by use of magnetic resonance imaging. Circulation.

[3] Dillman JR, Yarram SG, Hernandez RJ (2009). Imaging of pulmonary venous developmental anomalies. Am J Roentgenol.

[4] Porres DV, Morenza OP, Pallisa E Learning from the pulmonary veins, Radiographics 2013.

